# Pathogenic Germline Variants in BRCA1/2 and p53 Identified by Real-world Comprehensive Cancer Genome Profiling Tests in Asian Patients

**DOI:** 10.1158/2767-9764.CRC-23-0018

**Published:** 2023-11-14

**Authors:** Tomoyuki Satake, Shunsuke Kondo, Noriko Tanabe, Takaaki Mizuno, Yuki Katsuya, Jun Sato, Takafumi Koyama, Tatsuya Yoshida, Makoto Hirata, Noboru Yamamoto

**Affiliations:** 1 Department of Experimental Therapeutics, National Cancer Center Hospital, Tokyo, Japan.; 2 Department of Hepatobiliary and Pancreatic Oncology, National Cancer Center Hospital East, Kashiwa, Japan.; 3 Outpatient Treatment Center, National Cancer Center Hospital, Tokyo, Japan.; 4 Genetic Medicine and Services, National Cancer Center Hospital, Tokyo, Japan.

## Abstract

Cancer genome profiling (CGP) occasionally identifies pathogenic germline variants (PGV) in cancer susceptibility genes (CSG) as secondary findings. Here, we analyzed the prevalence and clinical characteristics of PGVs based on nationwide real-world data from CGP tests in Japan. We analyzed the genomic information and clinical characteristics of 23,928 patients with solid cancers who underwent either tumor-only (*n* = 20,189) or paired tumor-normal (*n* = 3,739) sequencing CGP tests between June 2019 and December 2021 using the comprehensive national database. We assigned clinical significance for all variants and highlighted the prevalence and characteristics of PGVs. Our primary analysis of the tumor-normal sequencing cohort revealed that 152 patients (4.1%) harbored PGVs in 15 CSGs. Among 783 germline variants, 113 were annotated as PGVs, 70 as benign variants, and 600 as variants of uncertain significance. The number of PGVs identified was highest in *BRCA1/2*, with 56, followed by *TP53*, with 18. PGVs were the most prevalent in ovarian and peritoneal cancers, including among cancer types common in Asia. In the tumor-only sequencing cohort, of the 5,184 pathogenic somatic variants across 26 CSGs, 784 (15.1%) were extracted according to the European Society for Medical Oncology recommendations for germline-focused tumor analysis. The prevalence of PGVs was similar to that previously reported in Europe and the United States. This is the largest analysis based on real-world tumor-normal sequencing tests in Asia. The more widespread use of the tumor-normal sequencing CGP test could be reasonable for evaluating PGVs.

**Significance:**

We analyzed real-world data from over 23,000 patients in Japan, revealing 4.1% harbored PGVs, particularly in *BRCA1/2* and *TP53*, in CSGs. It highlights the prevalence of PGVs in Asian populations and supports the broader adoption of tumor-normal sequencing CGP tests for PGV evaluation.

## Introduction

In clinical settings, massively parallel next-generation sequencing (NGS)-based cancer genome profiling (CGP) has become a routine practice for patients with advanced solid cancers worldwide for identifying clinically actionable somatic alterations for systemic therapy selection (1). Most commercial testing involves tumor-only sequencing, which can detect presumed germline pathogenic variants (PGPV). However, distinguishing somatic variants from germline variants in tumor-only sequencing data is difficult without the paired analysis of matched tumor-normal sequencing. Implementing a paired analysis in routine practice is limited by its cost- and effort-intensive coordination. Therefore, paired tumor-normal testing is rarely used outside the research setting; tumor-only sequencing is common (2, 3). Both tumor-only and tumor-normal sequencing CGP tests can potentially uncover a heritable predisposition to cancer, that is, the cancer susceptibility genes (CSG). A subset of variants identified in CSGs is pathogenic germline variants (PGV), referred to as secondary findings. PGVs can provide information about personal and familial cancer risks and guide patient care and cancer screening approaches. Previous studies have shown that PGVs are not rare and have a high expected prevalence in unselected cancer populations, including patients who do not meet the current clinical germline testing criteria. The prevalence of PGVs in paired tumor-normal sequencing assays ranges between 3.3% and 17.5% (4–8). The European Society for Medical Oncology (ESMO) recommends criteria for germline-focused tumor analysis and germline follow-up testing intended to optimize the identification of actionable PGVs in tumor sequencing (3). These criteria consider the gene, tumor type, a tumor arising age, and variant allele frequency (VAF) uncovered by initial tumor sequencing to determine which, if any, are recommended for follow-up.

In Japan, three types of NGS-based CGP tests, OncoGuide NCC Oncopanel System (NOP), FoundationOne CDx (F1CDx), and FoundationOne Liquid CDx (F1L), are reimbursed by the national health insurance system and implemented in routine oncological practice. F1CDx is a tumor-only genome profiling test commonly used in the United States, whereas NOP is a paired tumor-normal test for both tumor and normal (blood) DNA, uniquely implemented in Japan. Genomic information and clinical characteristics, including multiple cancers and family history, of patients who underwent CGP tests are collected at the Center for Cancer Genomics and Advanced Therapeutics (C-CAT), established in the National Cancer Center (9). The C-CAT maintains a central database that provides reports with genome annotation and information about genotype-matched therapy according to genomic alterations. Most studies on PGVs or PGPVs in CGP tests have been reported from Europe and the United States, but until now, there has been no comprehensive analysis from Asia (3–8). In this study, we analyzed the prevalence and clinical characteristics of PGVs based on nationwide real-world data obtained from CGP tests.

## Materials and Methods

### Patients

This study included 23,928 patients who received tissue-based CGP tests, NOP (*n* = 3,739), or F1CDx (*n* = 20,189) and had their genomic analysis and clinical data registered with the C-CAT from June 2019 to December 2021. All patients had advanced or recurrent cancers that were refractory to standard chemotherapy. Patients with NOP tests were our main cohort for germline variant analysis. Written informed consent, including permission for possible data use for research and registration for the C-CAT, was obtained from all patients before NGS-based CGP tests. This study was conducted in accordance with the Declaration of Helsinki, and this study was approved by the ethics committee of the National Cancer Center Hospital (no. 2020-067).

### NGS-based Cancer-gene Panel Test

NGS-based CGP tests (NOP or F1CDx) covered by the Japanese National Health Insurance System were selected at the discretion of the physician or patient. F1 L was not included in this study.

NOP, a paired tumor-normal test, analyzed tumor and germline DNAs of 114 cancer-associated genes, 12 fusion genes, and tumor mutation burden (TMB) and provided germline variants for 16 selected genes (*APC, BRCA1, BRCA2, MLH1, MSH2, PTEN, RB1, RET, SMAD4, STK11, TP53, TSC1,* and *VHL* included from the beginning; *NF1*, *PALB2,* and *SMARCB1* included from October 2020) from June 2019 to January 2021 (10). Germline findings were limited to single-nucleotide variants (SNV) and small insertions or deletions. NOP was revised in February 2021 and analyzed somatic and germline DNAs of 124 cancer-associated genes, 13 fusion genes, microsatellite instability (MSI), and TMB and provided germline variants for all 124 genes (11). Analysis of NOP was conducted by Sysmex, Inc. Genomic DNA was extracted from formalin-fixed paraffin-embedded (FFPE) tumor tissues (16 mm^2^ area and 50 µm thickness) with tumor concentration ratios ≥ 20% and peripheral blood (2 mL) cells using a QIAamp DNA FFPE Tissue kit (Qiagen) and a Maxwell RSC Blood DNA kit (Promega), respectively. The extracted DNA was quantified using a Qubit dsDNA BR Assay Kit (Thermo Fisher Scientific) and Qubit 3.0 Fluorometer (Thermo Fisher Scientific). Sequencing libraries were prepared from 50–800 ng DNA using the SureSelect XT reagent (Agilent Technologies) and KAPA Hyper Prep kit (KAPA Biosystems) and then analyzed on the Illumina MiSeq or NextSeq platform (Illumina) with 150 bp paired-end reads.

F1CDx analyzes only tumor DNAs for genomic alterations in 324 cancer-related genes (309 genes for nucleotide substitutions, small insertion/deletion mutations, and copy-number variation, and 36 genes for fusion), MSI, and TMB (12, 13). F1CDx requires FFPE tissues with an area ≥ 25 mm^2^, thickness of 40–50 µm, and tumor concentration ratios ≥ 20%. Analysis of F1CDx was conducted by Foundation Medicine, Inc. using NGS (HiSeq 4000, Illumina). F1CDx cannot directly identify germline variants. As the data of Asian SNPs are not incorporated into the F1CDx database, F1CDx cannot exclude SNPs unique to Asians.

### Genes for the Disclosure of CSGs to Patients

The genes for disclosing CSGs to patients are listed in the American College of Medical Genetics and Genomics (ACMG) recommendations for secondary findings, ESMO guidelines, National Comprehensive Cancer Network clinical practice guidelines, and the Japan Agency for Medical Research and Development (3, 14–16). Among these genes, 37 CSGs that were included for analysis in NOP or F1CDx were selected as subjects of this study (Supplementary Table S1). The CSGs included for analysis in NOP were changed before and after revision. Fifteen CSGs (*APC, BRCA1, BRCA2, MLH1, MSH2, NF1, PALB2, PTEN, RB1, RET, SMAD4, STK11, TP53, TSC1,* and *VHL*) were included in NOP before revision (NOP Ver.1), and an additional 11 CSGs (*ATM, BAP1, BARD1, CHEK2, MEN1, MSH6, NF2, PMS2, POLE, RAD51C*, and *TSC2*) were included after revision (NOP Ver.2; Supplementary Fig. S1). The “pathogenic” or “likely pathogenic” variants of these CSGs were defined as PGVs.

### Evaluation of Variant Pathogenicity

Sequencing data of the CGP tests were submitted to the C-CAT along with clinical data from each patient, including diagnostics, treatment, and outcome information. The C-CAT has constructed a cancer knowledge database (CKDB) optimized for Japan to assist decision-making (9). Although NOP and F1CDx have originally annotated reports, the C-CAT issues its reports based on the submitted data and CKDB. CKDB consists of four databases—marker database listing cancer genomic abnormalities; drug database listing approved drugs or drugs under clinical trials and their targets; evidence database consisting of public databases, including CIViC (Clinical Interpretation of Variants in Cancer), BRCA Exchange, ClinVar, and COSMIC (Catalogue of Somatic Mutations in Cancer); and a clinical trial database listing ongoing clinical trials in Japan. We evaluated the pathogenicity of CSGs based on C-CAT annotation. For further confirmation, all CSG variants were reassessed by genetic experts for pathogenicity based on ACMG and the Association for Molecular Pathology (AMP) guidelines (17).

### ESMO Recommendations for Germline-focused Tumor Analysis of Tumor-only CGP Tests (FoundationOne CDx)

F1CDx, a tumor-only CGP test, cannot directly detect germline variants. We evaluated CSGs with high actionability based on the ESMO recommendations (3). Germline-focused tumor analysis was restricted to variants classified as pathogenic/likely pathogenic based on ClinVar, except for benign SNPs with a threshold allele frequency of 0.01 or more in relevant databases, such as 1000 Genomes and the Integrative Japanese Genome Variation Database (iJGVD; refs. 18, 19). We focused our analysis of F1CDx on “high-actionability” CSGs in ESMO recommendations, defined as those of a level of actionability by which return of PGVs would be appropriate whether or not there is an established germline association between the gene and the tumor type. Germline-focused tumor analysis was limited to pathogenic/likely pathogenic variants (with VAF ≥30% for SNV and ≥20% for small insertion or deletion) and restricted to “on-tumor” as conferring predisposition to specific tumor types through prior research (*BAP1, FH, FLCN, NF1, POLE,* and *TP53*), early (<30 years) onset (*APC, RB1, NF1,* and *TP53*), and a high conversion ratio of somatic to germline variants [*BRCA1/2, BRIP1, MLH1, MSH2, MSH6, PALB2, PMS2, RAD51C, RAD51D, RET, SDHA, SDHB, SDHC, SDHD, TSC2, MUTYH,* and *VHL* (except for renal tumors)]. Filters with VAF ≥ 30% can cause misleading and should be used with caution, but we evaluated CSG variants based on the ESMO recommendations, which were established with the intent of avoiding excessive diversion of effort and resources towards “germline follow-up testing” of vast numbers of variants in tumor-only CGP tests.

### Statistical Analysis

Categorical variables were compared using Pearson *χ*^2^ test or Fisher exact test. The median values of the variables were compared using Kruskal–Wallis test. Statistical significance was set at *P* < 0.05. Statistical analysis was performed using EZR software version 1.38 (Saitama Medical Center, Jichi Medical University, Saitama, Japan; ref. 20). The current study adheres to the TRIPOD (Transparent Reporting of a Multivariable Prediction Model for Individual Prognosis or Diagnosis) guidelines for reporting (21).

### Data Availability

Raw data for this study were generated from C-CAT Research-Use Portal site (https://www.ncc.go.jp/en/c_cat/use/index.html). Institutional review at your institution and C-CAT data utilization review are required, but these data are publicly available. Derived data supporting the findings of this study are available from the corresponding author upon request.

## Results

### Cohort Characteristics

The characteristics of the enrolled patients are summarized in Table 1. In the paired tumor-normal test cohort, the mean age at CGP tests and first primary tumor diagnosis was 58.5 ± 15.2 and 56.6 ± 15.3 years, respectively. Of the patients, 51.3% were female, and most (94.4%) had Eastern Cooperative Oncology Group performance status scores of 0 or 1. A family history of cancer was observed in 66.8% of patients. The most frequent tumor types were pancreatic (18.3%), bowel (14.9%), breast (7.7%), biliary tract (7.5%), and esophagus/stomach (7.3%). Patient characteristics and frequent tumor types were similar for the tumor-only test cohort.

**TABLE 1:** Demographic and clinical characteristics of patients

	NOP		F1CDx	
Characteristics	*N* = 3,739		*N* = 20,189	
Sex				
Male	1,820	48.7%	9,510	47.1%
Female	1,919	51.3%	10,674	52.9%
Age at entry, years				
Mean (SD)	58.5	(±15.2)	58.7	(±15.4)
Age at diagnosis, years				
Mean (SD)	56.6	(±15.3)	56.8	(±15.5)
Age at diagnosis, years				
0–19	122	3.3%	663	3.3%
20–29	71	1.9%	343	1.7%
30–39	251	6.7%	926	4.6%
40–49	568	15.2%	2,706	13.4%
50–59	909	24.3%	4,446	22.0%
60–69	1,061	28.4%	5,717	28.3%
70–79	691	18.5%	4,827	23.9%
80–	66	1.8%	561	2.8%
Smoking history				
Yes	1,523	40.7%	8,024	39.7%
No	1,972	52.7%	10,749	53.2%
N/A	244	6.5%	1,416	7.0%
Alcohol drinking history				
Yes	458	12.3%	2,349	11.6%
No	2,791	74.7%	15,160	75.1%
N/A	490	13.1%	2,680	13.3%
ECOG-PS				
0	2,073	55.4%	11,768	58.3%
1	1,456	38.9%	6,819	33.8%
2	94	2.5%	507	2.5%
3	13	0.4%	99	0.5%
4	5	0.1%	26	0.1%
N/A	98	2.6%	970	4.8%
Multiple primary cancer				
Yes	329	8.8%	1,683	8.3%
No	3,319	88.8%	17,682	87.6%
N/A	91	2.4%	824	4.1%
Family history of cancer				
Yes	2,499	66.8%	12,791	63.4%
No	1039	27.8%	5,993	29.7%
N/A	201	5.4%	1,405	7.0%
Cancer type				
Adrenal gland	15	0.4%	74	0.4%
Ampulla of Vater	24	0.6%	140	0.7%
Biliary tract	279	7.5%	1,372	6.8%
Bladder/urinary tract	63	1.7%	350	1.7%
Bone	21	0.6%	211	1.1%
Bowel	558	14.9%	3,273	16.2%
Breast	286	7.7%	1,380	6.8%
Cervix	80	2.1%	654	3.2%
CNS/Brain	87	2.3%	824	4.1%
Esophagus/Stomach	273	7.3%	1,230	6.1%
Eye	11	0.3%	22	0.1%
Head and neck	104	2.8%	890	4.4%
Kidney	31	0.8%	151	0.8%
Liver	37	1.0%	195	1.0%
Lung	208	5.6%	1,120	5.6%
Other	137	3.7%	679	3.4%
Ovary/Fallopian tube	203	5.4%	1,301	6.4%
Pancreas	685	18.3%	1,975	9.8%
Penis	2	0.1%	16	0.1%
Peripheral nervous system	14	0.4%	101	0.5%
Peritoneum	33	0.9%	173	0.9%
Pleura	7	0.2%	81	0.4%
Prostate	97	2.6%	971	4.8%
Skin	99	2.7%	364	1.8%
Soft tissue	152	4.1%	1,103	5.5%
Testis	10	0.3%	40	0.2%
Thymus	39	1.0%	198	1.0%
Thyroid	27	0.7%	224	1.1%
Uterus	148	4.0%	1,031	5.1%
Vulva/vagina	9	0.2%	46	0.2%

### Frequency of Pathogenic Germline Variants in the Paired Tumor-normal Test Cohort

**FIGURE 1:**
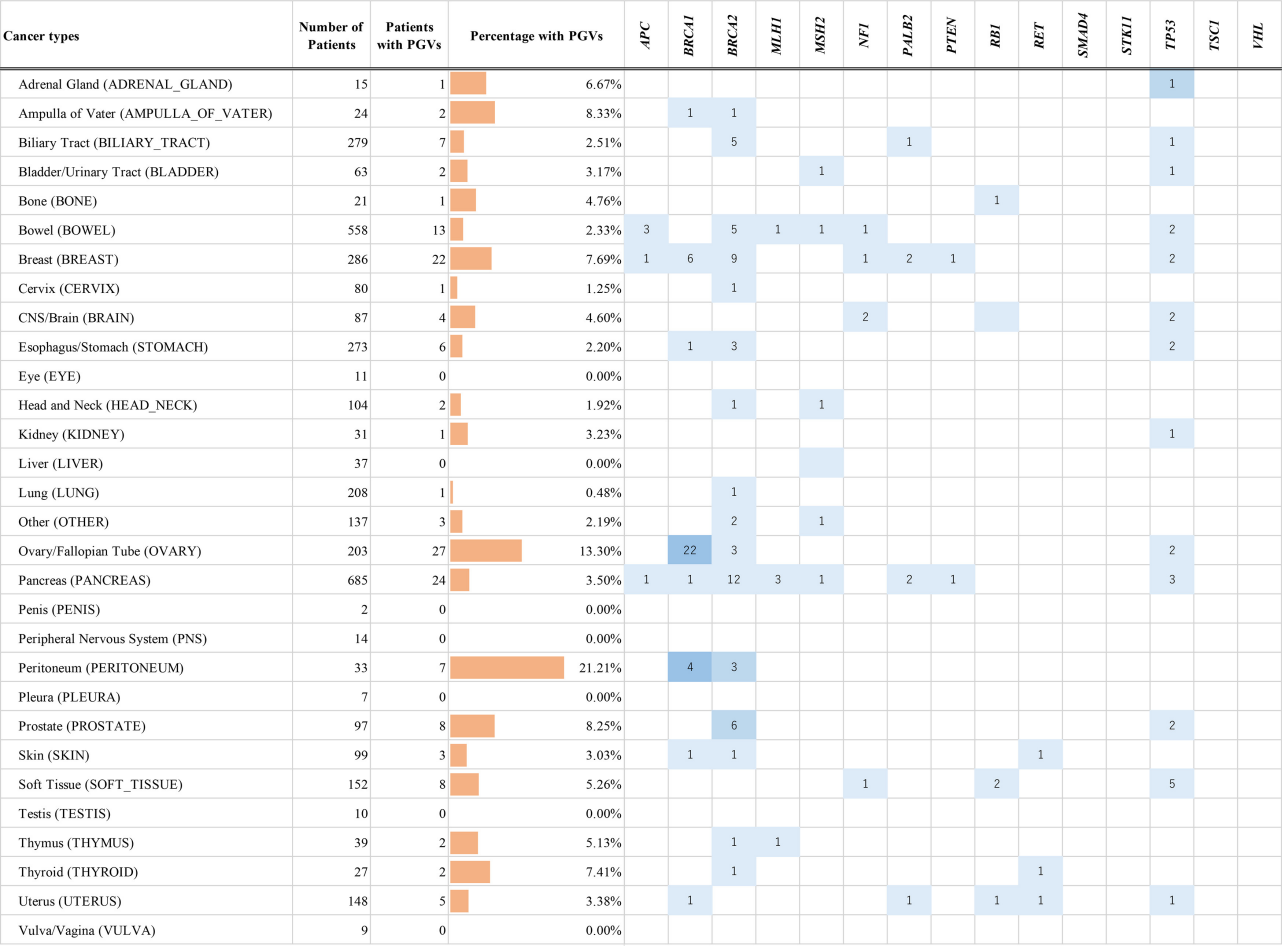
Prevalence of PGVs by cancer type and gene in the OncoGuide NOP. Analysis was performed using NOP data for the entire period, limited to only the cancer susceptibility genes subject to reporting of PGVs from NOP Ver.1. Shades of color correspond to the prevalence of PGVs in cancer types, with light to dark representing <5%, 5%–10%, and >10%.

The analysis of 15 CSGs tested throughout the study revealed that 152 patients (4.1%) had PGVs. Eight patients had PGVs in the 11 CSGs added to the germline analysis target in NOP Ver.2. PGVs were observed in several tumor types, and the number of identified PGVs was highest in *BRCA1*, *BRCA2*, and *TP53* (Fig. 1; Supplementary Table S2). PGVs were prevalent among cancers strongly associated with CSGs (breast and bowel) and others (esophagus/stomach, biliary tract, and ampulla of Vater). The frequencies of PGVs in the 15 CSGs were as follows: peritoneal (21.2%), ovary/fallopian tube (13.3%), prostate (8.3%), ampulla of Vater (8.3%), breast (7.7%), and thyroid cancer (7.4%). No PGVs were detected in *SMAD4, STK11, TSC1*, or *VHL*.

### Age Distribution of Patients with Pathogenic Germline Variants in the Paired Tumor-normal Test Cohort

**FIGURE 2:**
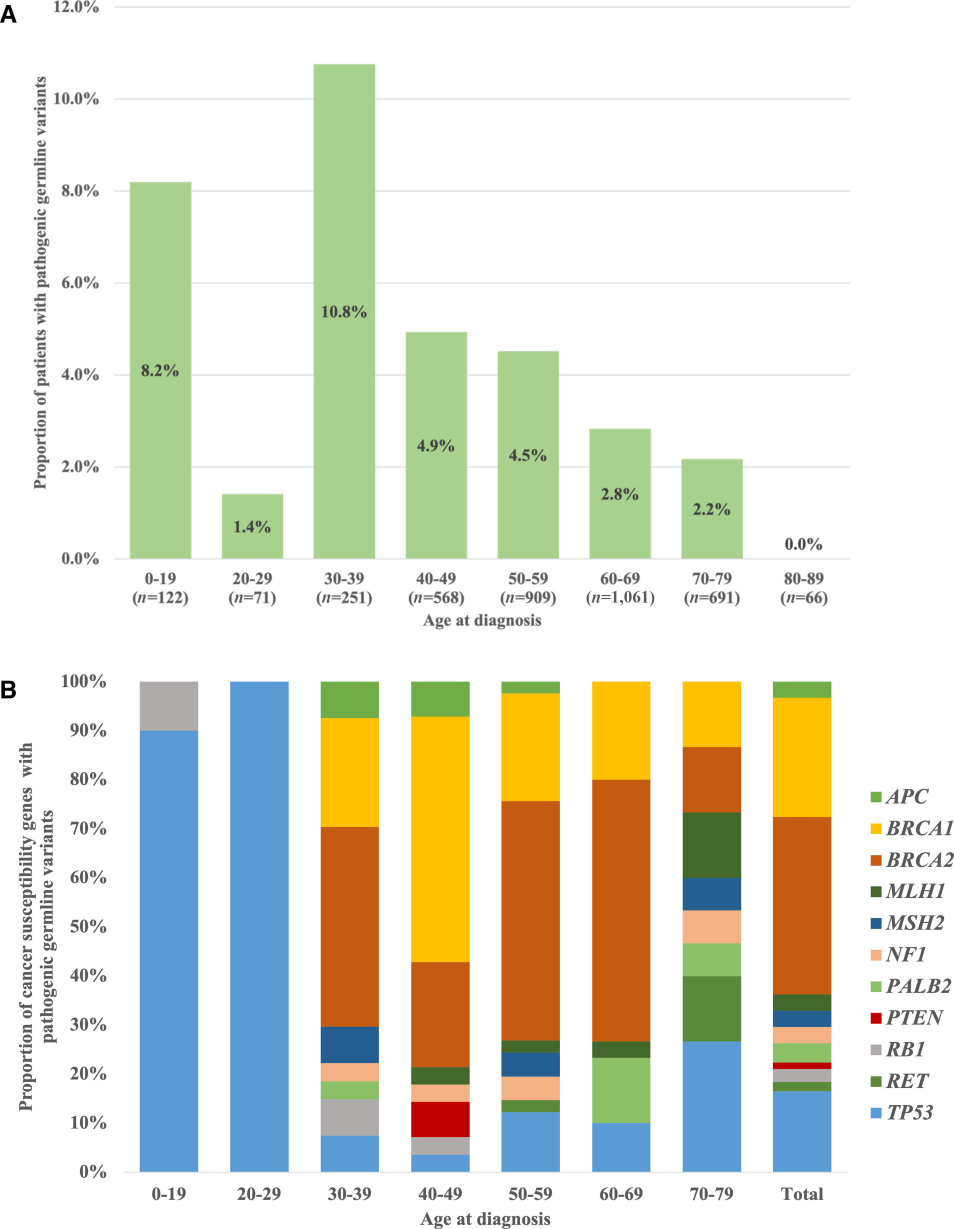
Proportion of patients (**A**) and CSGs (**B**) with PGVs by age group in the OncoGuide NOP. Analysis was performed using NOP data for the entire period, limited to only the CSGs subject to reporting of PGVs from NOP Ver.1.

We examined the impact of PGVs on age at tumor diagnosis by stratifying them into 10-year age groupings (Fig. 2). The proportion of patients with PGVs in each age group is shown in Fig. 2A. PGVs were found relatively frequently in children, adolescents, and young adults (AYA) under 40 years of age. In contrast, after 40 years of age, the proportion of PGVs decreased; the proportion was 2%–3% (45 patients) in individuals over 60 years of age. The CSGs with PGV and their proportion in each age group are shown in Fig. 2B. Pathogenic germline *BRCA1/2* variants were not found in patients under 30 years of age but were common PGVs in patients over 30 years of age, whereas 40% (10 patients) of *TP53* variants were detected in patients under 30 years of age. Various PGVs were observed in individuals over 70 years of age. Pathogenic germline *TP53* variants were found not only in the younger generation (≤40 years), which is one of the diagnostic criteria for Li-Fraumeni syndrome (LFS) but also in the older generation (>50 years; Supplementary Table S2). A few cases of low VAF (≤30%) in germline *TP53* variants were included.

### Germline Variants in the Paired Tumor-normal Test Cohort

**FIGURE 3:**
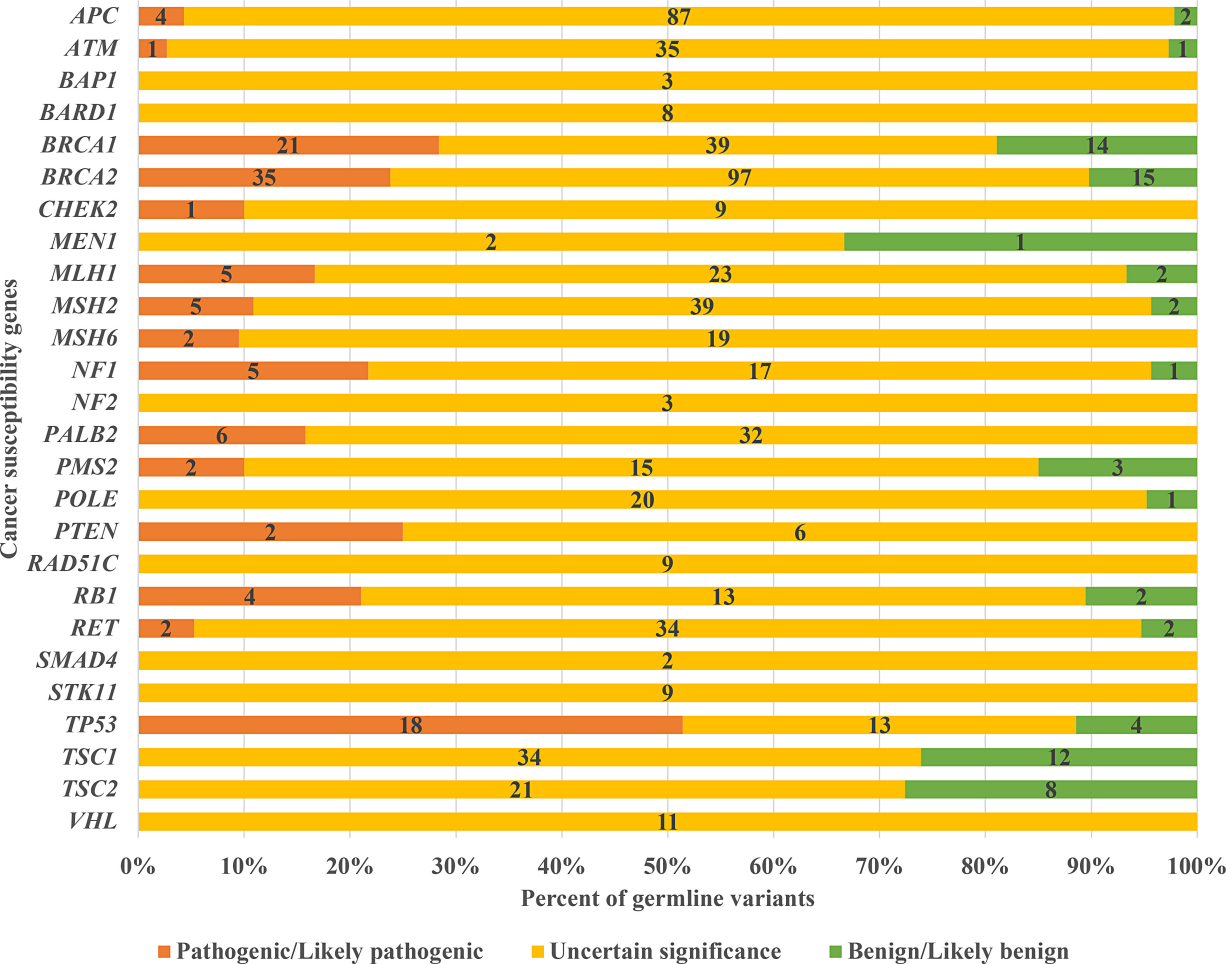
Distribution of germline variants categorized as pathogenic, benign, and of uncertain significance in the OncoGuide NOP. CSGs that were subject to PGV reporting in NOP Ver.2 were analyzed using only the NOP Ver.2 data, and CSGs that were subject to PGV reporting in NOP Ver.1 were analyzed using the NOP data for the entire period. Numbers in the graph indicate the number of variants.

Paired tumor-normal NOP tests for tumor and germline DNA detected 783 variants across the 26 CSGs. Among these variants, 113 were annotated as PGVs, 70 as benign, and 600 as variants of uncertain significance (VUS). We determined whether these variants were pathogenic based on CKDB, ClinVar, 1000 Genomes, iJGVD, and ACMG/AMP guidelines, and variants were assigned VUS if not listed in these databases. *TP53* was pathogenic in approximately half of the cases; however, other genes were frequently assigned as VUS. Even for *BRCA1*/2, the most common PGV, the proportion of VUS and benign tumors exceeded 70% (Fig. 3).

**FIGURE 4:**
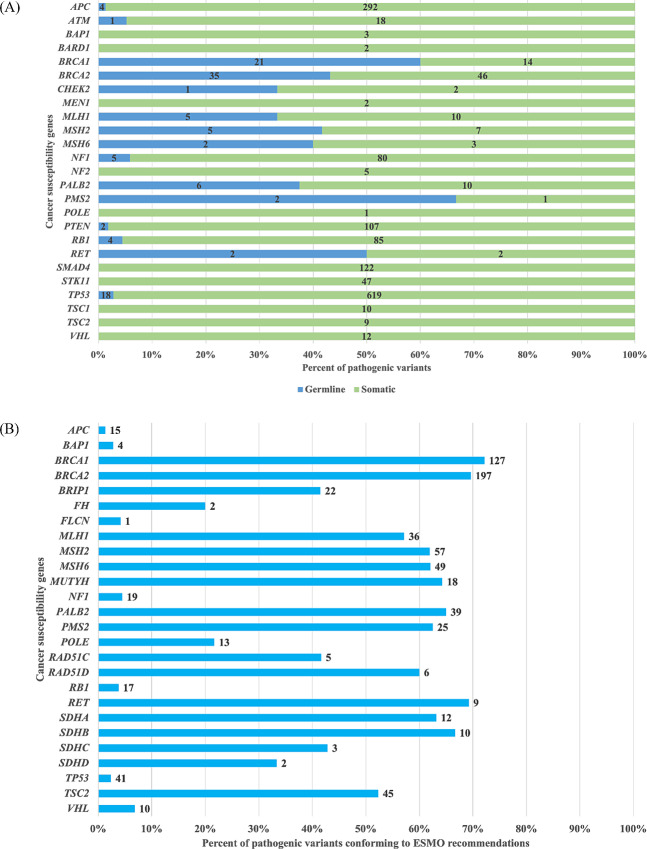
**A,** Distribution of germline and somatic variants categorized as pathogenic in the OncoGuide NOP. CSGs that were subject to PGV reporting in NOP Ver.2 were analyzed using only the NOP Ver.2 data, and CSGs that were subject to PGV reporting in NOP Ver.1 were analyzed using the NOP data for the entire period. Numbers in the graph indicate the number of variants. **B,** Proportion of pathogenic variants in FoundationOne CDx recommended for germline-focused analysis using European Society for Medical Oncology recommendations. Numbers in the graph indicate the number of variants.

Paired tumor-normal NOP tests detected 1,534 somatic variants across the 26 CSGs. Among these variants, 1,509 were annotated as PGVs, most frequently in *TP53*, *APC*, *SMAD4*, and *PTEN*. A total of 1,622 variants were annotated as PGVs. The ratio of somatic to germline pathogenic variants varied by gene (Fig. 4A). For example, *TP53* somatic variants were more common than germline—619 patients had somatic variants and 18 had germline variants. *APC, PTEN, RB1*, and *NF1* somatic variants were also more common, with a proportion of more than 90%. Clinically important variants in certain genes were of germline origin compared with others. For example, 14 patients had somatic and 21 patients had germline *BRCA1* variants, whereas 46 patients had somatic and 35 had germline *BRCA2* variants. *PALB2*, a gene related to the homologous recombination repair (HRR) pathway, and *MLH1*, *MSH2*, and *MSH6*, involved in the mismatch repair (MMR) pathway, also had germline variants at a relatively high ratio of 30%–40%.

### Pathogenic Variants Conforming to ESMO Recommendations in the Tumor-only Test Cohort

The tumor-only F1CDx test detected 5,184 somatic variants annotated as PGVs across 26 CSGs listed in the ESMO recommendations for germline-focused tumor analysis. Among these somatic variants, 784 (15.1%) met the criteria for the ESMO recommendations (Fig. 4B). For CSGs for which germline-focused tumor analysis was recommended only for specific cancer types and age groups, the proportion of variants meeting these recommendations was approximately 1%–4%, except for *POLE* (22%) and *FH* (20%). In comparison, for other CSGs having a high conversion ratio of somatic to germline variants, more than 40% of variants met the recommendations criteria. The variants of HRR-related genes (*BRCA1*, *BRCA2*, and *PALB2*) and MMR genes (*MLH1*, *MSH2*, and *MSH6*) met the recommendations at a particularly high percentage.

### Comparison Between Germline and Somatic Variants in Paired Tumor-normal Test and Variants Conforming to ESMO Recommendations in Tumor-only Test

A comparison of the proportion of PGVs in NOP and the proportion of pathogenic variants conforming to the ESMO recommendations in F1CDx for each of the 16 common CSGs revealed no major differences. *BRCA2* and *TSC2* had significantly higher rates of pathogenic variants conforming to the ESMO recommendations in F1CDx than the rate of PGVs in NOP (Supplementary Table S3).

For *BRCA1/2*, we also compared patient backgrounds and cancer types with somatic and germline variants in NOP and pathogenic variants in F1CDx, consistent with ESMO recommendations (Supplementary Table S4).

In NOP, 86% of *BRCA1* pathogenic germline variants were found in breast or ovarian cancer, including peritoneal cancer. *BRCA1* somatic variants were common in breast and ovarian cancers but occurred less frequently than germline variants. *BRCA1* somatic variants were more prevalent than germline variants in uterine cancers. *BRCA1* accounted for the majority of variants in F1CDx and was similarly observed in ovarian and breast cancers.

In contrast, in NOP, *BRCA2* germline variants were most common in pancreatic cancer, followed by breast, ovarian (including peritoneal), and prostate cancers, whereas *BRCA2* somatic variants were not found in ovarian cancer, were increased in gastrointestinal and prostate cancers, and were less common in breast and ovarian cancers than germline variants. Somatic variants were more common in males. In F1CDx, *BRCA2* mutations were more common in the prostate than in breast or pancreatic cancers.

In *BRCA1* and *BRCA2*, somatic variants were associated with a higher median patient age than germline variants. There were no significant differences in the presence or absence of family history and multiple cancers among variants in NOP and F1CDx.

### VAF for Pathogenic Germline Variants

We examined the distribution of non-tumor (peripheral blood) VAF for PGVs detected in NOP (Supplementary Fig. S2). The median non-tumor VAF was 46% (range, 10%–68%). Only nine of 160 (5.6%) PGVs, including *MLH1*, *PMS2*, *PALB2*, *RB1*, and *TP53*, had a VAF of less than 30%, and other PGVs were 30%–70%.

## Discussion

To the best of our knowledge, this is the largest nationwide analysis of real-world CGP data from patients with various advanced solid tumors. We observed PGVs across various CSGs, cancer types, and patient ages. We found that 4.1% of patients had PGVs. Although the frequency of PGVs was within the range previously reported by paired tumor-normal sequencing assays or hereditary genetic tests (4–8), it was relatively lower because the CSGs subjected to PGV analysis were different in each study. For example, a study by the MSK-IMPACT panel, which included 76 genes of clinical interest, reported a PGV prevalence of 17.5% (5); we restricted our primary analysis to 15 genes. A limitation of our study was that NOP Ver.1 lacked several important CSGs that are now considered actionable for breast and ovarian cancer in particular such as *MSH6*, *PMS2*, *RAD51C* and *RAD51D*, and NOP Ver.2 still did not include *SDHB*, *SDHC*, *SDHD*, and *MUTYH*, which were included in ACMG and ESMO recommendations (3, 10, 11, 14). Differences in cancer types, disease stage, patient age, and the number or characteristics of participating sites are other reasons for differences in the reported prevalence of PGVs. In Japan, CGP tests are reimbursed by national insurance only for those patients in whom malignant tumors progressed after standard of care; therefore, the cancer types for which CGP tests were performed differed from the incidence of each cancer type. However, our study has some advantages over previous studies. Most patients agree to have their genomic information and clinical characteristics submitted to the C-CAT, as they can receive annotation reports reviewed by the molecular tumor board (22). To date, most published studies have been based on data from a single or limited number of referral center sites; we cannot exclude the possibility of bias in patients referred to these specialized sites or enrolled in these studies, which may influence the overall results/detection of PGV tumor types. Our study used a national database of unbiased, comprehensive, and real-world data. In addition, our study highlights differences between races and cancer types. Most previous studies on PGVs in CGP tests are from Europe and the United States, with only a small percentage of Asian data included. In a single-institution Japanese study, Nagashima and colleagues (23) reported that germline driver mutations were detected in 9.2% of cases in 25 hereditary cancer genes, with 12.2% (1.1% of total) confirmed as pathogenic mutations. The prevalence of cancer types differs across regions, with some cancers being more common in Asia (24). The proportion of biliary tract and gastric cancers was higher in our study than in previous studies (4–8). A recent report by Momozawa and colleagues (25) showed that PGVs in *BRCA1* and/or *BRCA2* were associated with an increased risk of the biliary tract, gastric, and esophageal cancers, suggesting that the range of cancer types associated with PGVs in *BRCA1* and *BRCA2* was broader than that previously determined from the analyses of cohorts of largely European ancestry. Similarly, the prevalence of PGVs in CSGs may differ from that reported in Europe and the United States; our study can be an important reference for Asia. Our study reflects the current state of PGV detection using CGP tests in clinical practice based on comprehensive data from across Japan.

Our study also revealed a relationship between age and PGVs across cancer types. Previous studies have focused on a specific cancer type and reported the prevalence and distribution of PGVs in CSGs. For example, the prevalence of PGVs of selected CSGs was reported to be high in patients with breast cancer under the age of 40 years and in patients with prostate cancer under the age of 60 years, but it decreased with advancing age (26, 27). In contrast, in a cross-sectional analysis involving various cancer types, germline analysis following tumor sequencing showed that PGVs were modestly enriched in patients between the ages of 30 and 49 years but were prevalent across all age groups (28). Although there are few reports on the prevalence of PGVs in children, PGVs of genes with high or moderate penetrance were found in 8%–13% of patients with pediatric cancer (29–32). A meta-analysis suggests that PGVs in *BRCA1, BRCA2,* and MMR genes contribute to reduced penetrance to cancer risk in children and adolescents (33). Our study included patients of all cancer types and ages. The prevalence of PGVs in children was similar to that in previous reports. The tumor-normal NOP tests found no PGVs in *BRCA1*, *BRCA2*, *MLH1*, and *MSH2* in patients under 20 years of age, whereas the tumor-only F1CDx tests showed nine cases of *BRCA1* or *BRCA2* and seven cases of *MLH1* or *MSH2* with pathogenic variants. From our NOP data, it is still controversial whether *BRCA1, BRCA2,* and MMR genes are associated with an increased risk of cancer in children and adolescents, and further studies are needed. In adults, the prevalence of PGVs was highest in the AYA generation and decreased with age, unlike that in previous reports. Identifying hereditary cancer predisposition is highly relevant to the clinical care of young and old patients alike and has important implications for the medical guidance of their relatives.

According to ACMG recommendations, only “pathogenic” or “likely pathogenic” findings should be reported to patients (14). More than half of the germline variants identified in this study were classified as VUS, consistent with previous reports (5, 34). The clinical relevance of VUS remains unclear, and the high proportion of VUS identified using CGP tests raises concerns regarding their interpretation. However, these VUS results would likely have been reported in patients suspected of having an underlying hereditary cancer predisposition. Further investigation of VUS results, such as segregation analysis and functional studies, is necessary to provide additional evidence of pathogenicity (8). The limitation of our study is that tumor-normal NOP tests only report SNVs and small indels and do not detect large deletions and duplications. NOP test is not a validated germline panel, and therefore additional commercial multi-gene panel testing can reduce the VUS rate.

Both tumor-only F1CDx and tumor-normal NOP tests can be used under the Japanese health insurance system. An indirect comparison of the data distribution for both NOP-identified somatic and germline variants and F1CDx-identified variants requiring germline-focused analysis showed similar trends. Although the number of variants subjected to germline-focused analysis in F1CDx was higher than that in NOP, the ESMO recommendations were confirmed to be a useful guideline in the Asian cohort. The limitation of our study is that we did not analyze the same specimens for NOP and F1CDx, respectively, which might add selection bias in the comparison of the two.

Although the ESMO recommendations are useful, it should be noted that they are filtered by VAF, age, and cancer type. Heterozygous pathogenic variants are assumed to be germline origin when VAF is approximately 50%, with a range of 30%–70% commonly accepted (35). However, VAF can be misleading owing to tissue heterogeneity (purity of the analyzed sample) and tumor heterogeneity (existence of different clones within a tumor; ref. 36). Mandelker and colleagues (3) reported that a small number (3.5%) of PGVs could not be identified by the VAF filter for ESMO recommendations. Similarly, in our NOP data, 2.5% of PGVs had a low VAF of less than 20% in non-tumor (peripheral blood) samples. These variants with low VAF values may indicate postzygotic mosaicism or clonal hematopoiesis.

Regarding the age filter, our NOP data showed a small number of cases in which PGVs, including *TP53* and *APC*, were recognized, even in patients over 30 years old. For *TP53*, more than half and almost half of PGVs were detected in patients ages 30 and 50 years or older, respectively. *TP53* is known to cause LFS, a cancer predisposition syndrome. With the increasing use of NGS-based CGP tests, *TP53* germline genetic testing is frequently performed in individuals who do not meet the LFS genetic testing criteria, leading to the detection of *TP53* variant carriers associated with a less penetrant phenotype. The clinical significance and differences in phenotypes within the Li-Fraumeni spectrum are not yet clear. In our analyses, we were not able to differentiate low VAF between mosaicism and clonal hematopoiesis as no normal non blood cell analysis was performed. This is particularly important for TP53 in older patients. In terms of age, only 1 of 13 patients in the older generation (>45 years) had core LFS cancer, whereas nearly all of the patients in the younger generation (<45 years) had core LFS cancers. Y220C variant was found only in patients older than 60 years, all with VAF less than 30%. Y220C has been reported to be relatively more frequent in clonal hematopoiesis (37).

Tumor-normal NOP tests were more reliable than F1CDx for identifying PGVs. In Japan, both tumor-normal and tumor-only sequencing CGP tests are reimbursed by national insurance; therefore, it may be reasonable to normalize the use of tumor-normal sequencing tests.

Both tissue-based CGP and liquid-based CGP, such as F1L, have become increasingly popular in recent years; the ESMO recommendations are based on tissue-based data, and it is unclear whether these can be applied to liquid-based CGPs. Therefore, further research is required to evaluate whether ESMO recommendations can be extrapolated in their current form or whether new algorithms and guidelines are needed for germline-focused analysis based on liquid-based CGP data.

## Conclusions

In this study, we highlighted the prevalence of PGVs in CSGs based on nationwide, comprehensive data collected from CGP tests. PGVs were identified in 4.1% of the paired tumor-normal test cohort and contained many cancer types common in Asia. Although the NOP test contained limited CSGs, the prevalence of PGVs was generally similar to that previously reported in Europe and the United States. To the best of our knowledge, this is the largest analysis based on real-world tumor-normal sequencing tests in Asia. The ESMO recommendations might be useful for identifying the variants required for germline-focused analysis in tumor-only sequencing tests; however, more widespread use of the tumor-normal sequencing CGP test could be recommended for evaluating PGVs.

## Supplementary Material

Table S1 List of potentially actionable cancer susceptibility genes (CSGs)

Table S2 Detailed information about patients with BRCA1/2 and TP53 pathogenic germline variants

Table S3 Comparison of pathogenic germline variants

Table S4 Characteristics of patients with BRCA1/2 variants

Figure S1 The time-related changes in the OncoGuideTM NCC oncopanel system

Figure S2 Distribution of variant allele frequency
