# Supplementary material for: Pathogenic Germline Variants in BRCA1/2 and p53 Identified by Real-world Comprehensive Cancer Genome Profiling Tests in Asian Patients
Source: Cancer Res Commun. 2023 Nov 14;3(11):2302–11. doi: 10.1158/2767-9764.CRC-23-0018 (PMC10644847; doi:10.1158/2767-9764.CRC-23-0018)
Supplement: Table S1 — List of potentially actionable cancer susceptibility genes (CSGs) [file crc-23-0018-s01.docx]

**Supplementary Table S1.**

List of potentially actionable cancer susceptibility genes (CSGs) selected in this study

◎ Recommendation in principle; ◯ Recommendation in case with ‘on-tumor’ as conferring predisposition to specific tumor types or early (< 30 years) onset; ✓ Inclusion in the panel; ※ Inclusion for germline mutation analysis in the revised panel

| **List of potentially actionable CSGs** | **ESMO recommendation** | **NOP** | **F1CDx** |
| --- | --- | --- | --- |
| *APC* | ◯ | ✓ | ✓ |
| *ATM* |  | ※ | ✓ |
| *BAP1* | ◯ | ※ | ✓ |
| *BARD1* |  | ※ | ✓ |
| *BRCA1* | ◎ | ✓ | ✓ |
| *BRCA2* | ◎ | ✓ | ✓ |
| *BRIP1* | ◎ |  | ✓ |
| *CDH1* |  |  | ✓ |
| *CHEK2* |  | ※ | ✓ |
| *FH* | ◯ |  | ✓ |
| *FLCN* | ◯ |  | ✓ |
| *MEN1* |  | ※ | ✓ |
| *MLH1* | ◎ | ✓ | ✓ |
| *MSH2* | ◎ | ✓ | ✓ |
| *MSH6* | ◎ | ※ | ✓ |
| *MUTYH* | ◎ |  | ✓ |
| *NF1* | ◯ | ✓ | ✓ |
| *NF2* |  | ※ | ✓ |
| *PALB2* | ◎ | ✓ | ✓ |
| *PMS2* | ◎ | ※ | ✓ |
| *POLE* | ◯ | ※ | ✓ |
| *PTEN* |  | ✓ | ✓ |
| *RAD51C* | ◎ | ※ | ✓ |
| *RAD51D* | ◎ |  | ✓ |
| *RB1* | ◯ | ✓ | ✓ |
| *RET* | ◎ | ✓ | ✓ |
| *SDHA* | ◎ |  | ✓ |
| *SDHB* | ◎ |  | ✓ |
| *SDHC* | ◎ |  | ✓ |
| *SDHD* | ◎ |  | ✓ |
| *SMAD4* |  | ✓ | ✓ |
| *STK11* |  | ✓ | ✓ |
| *TP53* | ◯ | ✓ | ✓ |
| *TSC1* |  | ✓ | ✓ |
| *TSC2* | ◎ | ※ | ✓ |
| *VHL* | ◯ | ✓ | ✓ |
| *WT1* |  |  | ✓ |
