# Supplementary material for: Pathogenic Germline Variants in BRCA1/2 and p53 Identified by Real-world Comprehensive Cancer Genome Profiling Tests in Asian Patients
Source: Cancer Res Commun. 2023 Nov 14;3(11):2302–11. doi: 10.1158/2767-9764.CRC-23-0018 (PMC10644847; doi:10.1158/2767-9764.CRC-23-0018)
Supplement: Table S2 — Detailed information about patients with BRCA1/2 and TP53 pathogenic germline variants [file crc-23-0018-s02.docx]

**Supplementary Table S2.**

Detailed information about patients with *BRCA1/2* and *TP53* pathogenic germline variants

| **Patients** | **Cancer type** | **Age** | **Sex** | **Gene** | **Pathogenic variant** | **VAF** | **ClinVar** |
| --- | --- | --- | --- | --- | --- | --- | --- |
| 1 | Breast | 34 | F | *BRCA1* | L63* | 0.42 | Pathogenic |
| 2 | Ovary/Fallopian Tube | 36 | F | *BRCA1* | L63* | 0.47 | Pathogenic |
| 3 | Ovary/Fallopian Tube | 37 | F | *BRCA1* | L63* | 0.49 | Pathogenic |
| 4 | Ovary/Fallopian Tube | 38 | F | *BRCA1* | D1169fs*1 | 0.45 | Pathogenic |
| 5 | Ovary/Fallopian Tube | 39 | F | *BRCA1* | E1148fs*7 | 0.46 | Pathogenic |
| 6 | Ovary/Fallopian Tube | 39 | F | *BRCA1* | c.4485-2A>G | 0.42 | Pathogenic/Likely pathogenic​ |
| 7 | Breast | 40 | F | *BRCA1* | E797fs*3 | 0.42 | Pathogenic |
| 8 | Ovary/Fallopian Tube | 40 | F | *BRCA1* | L63* | 0.47 | Pathogenic |
| 9 | Ovary/Fallopian Tube | 40 | F | *BRCA1* | N1355fs*10 | 0.45 | Pathogenic |
| 10 | Ovary/Fallopian Tube | 42 | F | *BRCA1* | Q1447fs*16 | 0.38 | Pathogenic |
| 11 | Ovary/Fallopian Tube | 42 | F | *BRCA1* | H692fs*19 | 0.38 | Pathogenic |
| 12 | Ovary/Fallopian Tube | 44 | F | *BRCA1* | L1133fs*7 | 0.39 | Pathogenic |
| 13 | Peritoneum | 44 | F | *BRCA1* | K654fs*47 | 0.46 | Pathogenic |
| 14 | Breast | 45 | F | *BRCA1* | D1169fs*1 | 0.40 | Pathogenic |
| 15 | Ovary/Fallopian Tube | 46 | F | *BRCA1* | L1216* | 0.48 | Pathogenic |
| 16 | Ovary/Fallopian Tube | 47 | F | *BRCA1* | L63* | 0.48 | Pathogenic |
| 17 | Ovary/Fallopian Tube | 47 | F | *BRCA1* | N1215fs*3 | 0.47 | Pathogenic |
| 18 | Ovary/Fallopian Tube | 47 | F | *BRCA1* | K654fs*47 | 0.34 | Pathogenic |
| 19 | Breast | 48 | F | *BRCA1* | N838fs*8 | 0.50 | Pathogenic |
| 20 | Ovary/Fallopian Tube | 49 | F | *BRCA1* | L63* | 0.45 | Pathogenic |
| 21 | Ovary/Fallopian Tube | 50 | F | *BRCA1* | Q934* | 0.50 | Pathogenic |
| 22 | Breast | 51 | F | *BRCA1* | Q317* | 0.45 | Pathogenic |
| 23 | Ovary/Fallopian Tube | 52 | F | *BRCA1* | S896fs*104 | 0.44 | Pathogenic |
| 24 | Breast | 53 | F | *BRCA1* | L63* | 0.51 | Pathogenic |
| 25 | Peritoneum | 54 | F | *BRCA1* | Q1323* | 0.46 | Pathogenic |
| 26 | Ovary/Fallopian Tube | 55 | F | *BRCA1* | L63* | 0.41 | Pathogenic |
| 27 | Uterus | 56 | F | *BRCA1* | E1148fs*7 | 0.48 | Pathogenic |
| 28 | Ovary/Fallopian Tube | 59 | F | *BRCA1* | L63* | 0.45 | Pathogenic |
| 29 | Pancreas | 59 | F | *BRCA1* | S157fs*1 | 0.49 | Pathogenic |
| 30 | Ovary/Fallopian Tube | 60 | F | *BRCA1* | Q81* | 0.48 | Pathogenic |
| 31 | Ovary/Fallopian Tube | 61 | F | *BRCA1* | F107fs*12 | 0.44 | Pathogenic |
| 32 | Esophagus/Stomach | 62 | M | *BRCA1* | L63* | 0.48 | Pathogenic |
| 33 | Peritoneum | 63 | F | *BRCA1* | Y1853C | 0.48 | Pathogenic/Likely pathogenic​ |
| 34 | Ovary/Fallopian Tube | 65 | F | *BRCA1* | E1148fs*7 | 0.37 | Pathogenic |
| 35 | Peritoneum | 67 | F | *BRCA1* | L63* | 0.48 | Pathogenic |
| 36 | Skin | 72 | F | *BRCA1* | E1148fs*7 | 0.48 | Pathogenic |
| 37 | Ampulla of Vater | 76 | F | *BRCA1* | H692fs*19 | 0.43 | Pathogenic |
| 38 | Breast | 32 | F | *BRCA2* | R2318* | 0.45 | Pathogenic |
| 39 | Breast | 33 | F | *BRCA2* | T3033fs*11 | 0.46 | Pathogenic |
| 40 | Bowel | 34 | F | *BRCA2* | R2336H | 0.52 | Pathogenic |
| 41 | Pancreas | 35 | F | *BRCA2* | N2135fs*3 | 0.37 | Pathogenic |
| 42 | Biliary Tract | 36 | M | *BRCA2* | I1859fs*3 | 0.50 | Pathogenic |
| 43 | Thymus | 36 | M | *BRCA2* | R2318* | 0.45 | Pathogenic |
| 44 | Bowel | 37 | F | *BRCA2* | K467* | 0.55 | Pathogenic |
| 45 | Breast | 38 | F | *BRCA2* | T3033fs*11 | 0.51 | Pathogenic |
| 46 | Breast | 39 | F | *BRCA2* | I1859fs*3 | 0.45 | Pathogenic |
| 47 | Other | 39 | F | *BRCA2* | I1258fs*1 | 0.39 | Pathogenic |
| 48 | Biliary Tract | 39 | F | *BRCA2* | I332fs*17 | 0.39 | Pathogenic |
| 49 | Ovary/Fallopian Tube | 46 | F | *BRCA2* | A938fs*21 | 0.42 | Pathogenic |
| 50 | Bowel | 47 | F | *BRCA2* | c.8487+1G>A | 0.44 | Pathogenic |
| 51 | Pancreas | 47 | M | *BRCA2* | S1882* | 0.46 | Pathogenic |
| 52 | Head and Neck | 47 | M | *BRCA2* | I605fs*11 | 0.46 | Pathogenic |
| 53 | Thyroid | 49 | M | *BRCA2* | N2135fs*3 | 0.42 | Pathogenic |
| 54 | Prostate | 49 | M | *BRCA2* | V1447fs*1 | 0.36 | Pathogenic |
| 55 | Breast | 49 | F | *BRCA2* | c.631+1G>C | 0.43 | Pathogenic |
| 56 | Breast | 50 | F | *BRCA2* | R2602T | 0.48 | Pathogenic |
| 57 | Ovary/Fallopian Tube | 50 | F | *BRCA2* | R2318* | 0.39 | Pathogenic |
| 58 | Biliary Tract | 50 | M | *BRCA2* | R2112fs*16 | 0.47 | Pathogenic |
| 59 | Skin | 50 | M | *BRCA2* | Q3126* | 0.46 | Pathogenic |
| 60 | Esophagus/Stomach | 51 | F | *BRCA2* | R2318* | 0.46 | Pathogenic |
| 61 | Breast | 51 | F | *BRCA2* | R2318* | 0.48 | Pathogenic |
| 62 | Pancreas | 53 | M | *BRCA2* | E1550fs*4 | 0.41 | Pathogenic |
| 63 | Bowel | 54 | F | *BRCA2* | R2318* | 0.45 | Pathogenic |
| 64 | Pancreas | 54 | F | *BRCA2* | Y2154* | 0.45 | Pathogenic |
| 65 | Other | 54 | M | *BRCA2* | S1882* | 0.42 | Pathogenic |
| 66 | Prostate | 54 | M | *BRCA2* | I1859fs*3 | 0.46 | Pathogenic |
| 67 | Peritoneum | 54 | F | *BRCA2* | R2318* | 0.45 | Pathogenic |
| 68 | Lung | 54 | M | *BRCA2* | C1591fs*1 | 0.39 | Pathogenic |
| 69 | Pancreas | 55 | F | *BRCA2* | c.517-2A>G | 0.44 | Pathogenic |
| 70 | Cervix | 56 | F | *BRCA2* | S1079fs*8 | 0.57 | Pathogenic |
| 71 | Ovary/Fallopian Tube | 57 | F | *BRCA2* | D1177fs*19 | 0.42 | Pathogenic |
| 72 | Pancreas | 57 | F | *BRCA2* | S1262* | 0.31 | Pathogenic |
| 73 | Biliary Tract | 57 | M | *BRCA2* | c.8488-1G>A | 0.46 | Pathogenic |
| 74 | Breast | 59 | F | *BRCA2* | N1544fs*24 | 0.46 | Pathogenic |
| 75 | Peritoneum | 61 | F | *BRCA2* | T1317fs*2 | 0.49 | - |
| 76 | Biliary Tract | 62 | M | *BRCA2* | P3039P | 0.46 | Pathogenic |
| 77 | Pancreas | 62 | F | *BRCA2* | E1299* | 0.35 | - |
| 78 | Breast | 62 | M | *BRCA2* | Q2435* | 0.42 | Pathogenic |
| 79 | Prostate | 62 | M | *BRCA2* | K467* | 0.49 | Pathogenic |
| 80 | Pancreas | 63 | F | *BRCA2* | R2318* | 0.35 | Pathogenic |
| 81 | Esophagus/Stomach | 63 | M | *BRCA2* | V220fs*4 | 0.43 | Pathogenic |
| 82 | Pancreas | 64 | M | *BRCA2* | I1859fs*3 | 0.44 | Pathogenic |
| 83 | Ampulla of Vater | 65 | F | *BRCA2* | c.632-1G>A | 0.44 | Pathogenic |
| 84 | Esophagus/Stomach | 66 | F | *BRCA2* | C2256* | 0.49 | Pathogenic |
| 85 | Prostate | 66 | M | *BRCA2* | K1828fs*4 | 0.48 | Pathogenic |
| 86 | Pancreas | 66 | M | *BRCA2* | S2835* | 0.46 | Pathogenic |
| 87 | Peritoneum | 67 | F | *BRCA2* | R2318* | 0.59 | Pathogenic |
| 88 | Prostate | 69 | M | *BRCA2* | R2318* | 0.36 | Pathogenic |
| 89 | Prostate | 69 | M | *BRCA2* | R2318* | 0.47 | Pathogenic |
| 90 | Bowel | 69 | M | *BRCA2* | I1859fs*3 | 0.51 | Pathogenic |
| 91 | Pancreas | 70 | F | *BRCA2* | I1859fs*3 | 0.45 | Pathogenic |
| 92 | Pancreas | 75 | F | *BRCA2* | S2835* | 0.40 | Pathogenic |
| 93 | Soft Tissue | 1 | M | *TP53* | R248Q | 0.46 | Pathogenic |
| 94 | Adrenal Gland | 3 | F | *TP53* | G245S | 0.48 | Pathogenic |
| 95 | Soft Tissue | 3 | M | *TP53* | R282W | 0.47 | Pathogenic/Likely pathogenic​ |
| 96 | Soft Tissue | 4 | F | *TP53* | V73fs*50 | 0.42 | Pathogenic |
| 97 | Soft Tissue | 5 | M | *TP53* | K132E | 0.47 | Pathogenic/Likely pathogenic​ |
| 98 | Kidney | 7 | F | *TP53* | R273C | 0.47 | Pathogenic/Likely pathogenic​ |
| 99 | CNS/Brain | 9 | F | *TP53* | G245S | 0.47 | Pathogenic |
| 100 | Ovary/Fallopian Tube | 11 | F | *TP53* | R248Q | 0.46 | Pathogenic |
| 101 | Soft Tissue | 12 | F | *TP53* | R282W | 0.53 | Pathogenic/Likely pathogenic​ |
| 102 | Breast | 24 | F | *TP53* | R175H | 0.48 | Pathogenic |
| 103 | Uterus | 31 | F | *TP53* | R273H | 0.39 | Pathogenic |
| 104 | CNS/Brain | 32 | M | *TP53* | H214R | 0.51 | Likely pathogenic​ |
| 105 | Ovary/Fallopian Tube | 46 | F | *TP53* | R175H | 0.36 | Pathogenic |
| 106 | Biliary Tract | 50 | F | *TP53* | C242Y | 0.47 | Pathogenic |
| 107 | Pancreas | 51 | M | *TP53* | T86M | 0.50 | Likely pathogenic​ |
| 108 | Esophagus/Stomach | 55 | M | *TP53* | c.559+2T>G | 0.29 | - |
| 109 | Pancreas | 55 | F | *TP53* | R282P | 0.48 | Pathogenic/Likely pathogenic​ |
| 110 | Breast | 56 | F | *TP53* | R248W | 0.47 | Pathogenic |
| 111 | Bowel | 56 | F | *TP53* | M246V | 0.47 | Pathogenic |
| 112 | Bowel | 63 | F | *TP53* | W91fs*32 | 0.68 | Pathogenic |
| 113 | Prostate | 66 | M | *TP53* | Y220C | 0.27 | Pathogenic |
| 114 | Esophagus/Stomach | 68 | M | *TP53* | Y220C | 0.10 | Pathogenic |
| 115 | Pancreas | 70 | M | *TP53* | Y220C | 0.12 | Pathogenic |
| 116 | Prostate | 73 | M | *TP53* | Y220C | 0.11 | Pathogenic |
| 117 | Bladder/Urinary Tract | 75 | M | *TP53* | I195T | 0.46 | Pathogenic |
