# Supplementary material for: Pathogenic Germline Variants in BRCA1/2 and p53 Identified by Real-world Comprehensive Cancer Genome Profiling Tests in Asian Patients
Source: Cancer Res Commun. 2023 Nov 14;3(11):2302–11. doi: 10.1158/2767-9764.CRC-23-0018 (PMC10644847; doi:10.1158/2767-9764.CRC-23-0018)
Supplement: Table S3 — Comparison of pathogenic germline variants [file crc-23-0018-s03.docx]

**Supplementary Table S3.**

Comparison of pathogenic germline variants in the OncoGuide^TM^ NCC oncopanel system and pathogenic variants conforming to European Society for Medical Oncology recommendations in FoundationOne® CDx

| **Gene** | **F1CDx pathogenic variants** | **ESMO recommendation variants** | **ESMO recommendation (%)** | **NOP pathogenic variants** | **NOP PGVs** | **NOP PGVs (%)** | ***P-*value** |
| --- | --- | --- | --- | --- | --- | --- | --- |
| *APC* | 1148 | 15 | 1.3% | 296 | 4 | 1.4% | 1 |
| *BAP1* | 144 | 4 | 2.8% | 3 | 0 | 0.0% | 1 |
| *BRCA1* | 176 | 127 | 72.2% | 35 | 21 | 60.0% | 0.161 |
| *BRCA2* | 283 | 197 | 69.6% | 81 | 35 | 43.2% | <0.001 |
| *MLH1* | 63 | 36 | 57.1% | 15 | 5 | 33.3% | 0.150 |
| *MSH2* | 92 | 57 | 62.0% | 12 | 5 | 41.7% | 0.218 |
| *MSH6* | 79 | 49 | 62.0% | 5 | 2 | 40.0% | 0.377 |
| *NF1* | 426 | 19 | 4.5% | 85 | 5 | 5.9% | 0.574 |
| *PALB2* | 60 | 39 | 65.0% | 16 | 6 | 37.5% | 0.084 |
| *PMS2* | 40 | 25 | 62.5% | 3 | 2 | 66.7% | 1 |
| *POLE* | 60 | 13 | 21.7% | 1 | 0 | 0.0% | 1 |
| *RB1* | 449 | 17 | 3.8% | 89 | 4 | 4.5% | 0.764 |
| *RET* | 13 | 9 | 69.2% | 4 | 2 | 50.0% | 0.584 |
| *TP53* | 1734 | 41 | 2.4% | 637 | 18 | 2.8% | 0.552 |
| *TSC2* | 86 | 45 | 52.3% | 9 | 0 | 0.0% | 0.003 |
| *VHL* | 147 | 10 | 6.8% | 12 | 0 | 0.0% | 1 |
