# Supplementary material for: Pathogenic Germline Variants in BRCA1/2 and p53 Identified by Real-world Comprehensive Cancer Genome Profiling Tests in Asian Patients
Source: Cancer Res Commun. 2023 Nov 14;3(11):2302–11. doi: 10.1158/2767-9764.CRC-23-0018 (PMC10644847; doi:10.1158/2767-9764.CRC-23-0018)
Supplement: Table S4 — Characteristics of patients with BRCA1/2 variants [file crc-23-0018-s04.docx]

**Supplementary Table S4.**

Characteristics of patients with *BRCA1/2* variants

| **Characteristics** | ***BRCA1*** | | | | | | ***P*-value** | ***BRCA2*** | | | | | | ***P*-value** |
| --- | --- | --- | --- | --- | --- | --- | --- | --- | --- | --- | --- | --- | --- | --- |
|  | **NOP** | | | | **F1CDx** | |  | **NOP** | | | | **F1CDx** | |  |
|  | **Germline** | | **Somatic** | | **ESMO recommendation** | |  | **Germline** | | **Somatic** | | **ESMO recommendation** | |  |
|  | ***N* = 37** | | ***N =* 18** | | ***N* = 242** | |  | ***N* = 55** | | ***N* = 51** | | ***N* = 425** | |  |
| Sex |  |  |  |  |  |  | 0.011 |  |  |  |  |  |  | 0.018 |
| Male | 1 | 2.7% | 5 | 27.8% | 46 | 19.0% |  | 23 | 41.8% | 32 | 62.7% | 203 | 47.8% |  |
| Female | 36 | 97.3% | 13 | 72.2% | 196 | 81.0% |  | 32 | 58.2% | 19 | 37.3% | 222 | 52.2% |  |
| Age at diagnosis, years |  |  |  |  |  |  | 0.051 |  |  |  |  |  |  | 0.017 |
| Median (range) | 48 | (34-76) | 55.5 | (34-73) | 53 | (7-89) |  | 54 | (32-75) | 62 | (17-84) | 59 | (0-91) |  |
| Age at diagnosis, years |  |  |  |  |  |  |  |  |  |  |  |  |  |  |
| 0–19 | 0 | 0.0% | 0 | 0.0% | 2 | 0.8% |  | 0 | 0.0% | 1 | 2.0% | 7 | 1.6% |  |
| 20–29 | 0 | 0.0% | 0 | 0.0% | 4 | 1.7% |  | 0 | 0.0% | 0 | 0.0% | 9 | 2.1% |  |
| 30–39 | 6 | 16.2% | 1 | 5.6% | 12 | 5.0% |  | 11 | 0.2% | 4 | 7.8% | 37 | 8.7% |  |
| 40–49 | 14 | 37.8% | 4 | 22.2% | 62 | 25.6% |  | 7 | 12.7% | 5 | 9.8% | 73 | 17.2% |  |
| 50–59 | 9 | 24.3% | 7 | 38.9% | 86 | 35.5% |  | 19 | 34.5% | 12 | 23.5% | 98 | 23.1% |  |
| 60–69 | 6 | 16.2% | 2 | 11.1% | 43 | 17.8% |  | 16 | 29.1% | 16 | 31.4% | 128 | 30.1% |  |
| 70–79 | 2 | 5.4% | 4 | 22.2% | 28 | 11.6% |  | 2 | 3.6% | 10 | 19.6% | 64 | 15.1% |  |
| 80– | 0 | 0.0% | 0 | 0.0% | 5 | 2.1% |  | 0 | 0.0% | 3 | 5.9% | 9 | 2.1% |  |
| Multiple primary cancer |  |  |  |  |  |  | 0.601 |  |  |  |  |  |  | 0.070 |
| Yes | 4 | 10.8% | 2 | 11.1% | 37 | 15.3% |  | 2 | 3.6% | 10 | 19.6% | 48 | 11.3% |  |
| No | 33 | 89.2% | 16 | 88.9% | 192 | 79.3% |  | 53 | 96.4% | 40 | 78.4% | 365 | 85.9% |  |
| N/A | 0 | 0.0% | 0 | 0.0% | 13 | 5.4% |  | 0 | 0.0% | 1 | 2.0% | 12 | 2.8% |  |
| Family history of cancer |  |  |  |  |  |  | 0.888 |  |  |  |  |  |  | 0.294 |
| Yes | 30 | 81.1% | 13 | 72.2% | 175 | 72.3% |  | 47 | 85.5% | 36 | 70.6% | 325 | 76.5% |  |
| No | 5 | 13.5% | 4 | 22.2% | 47 | 19.4% |  | 7 | 12.7% | 10 | 19.6% | 79 | 18.6% |  |
| N/A | 2 | 5.4% | 1 | 5.6% | 20 | 8.3% |  | 1 | 1.8% | 5 | 9.8% | 21 | 4.9% |  |
| Cancer types |  |  |  |  |  |  | 0.029 |  |  |  |  |  |  | 0.014 |
| Adrenal gland | 0 | 0.0% | 0 | 0.0% | 0 | 0.0% |  | 0 | 0.0% | 0 | 0.0% | 1 | 0.2% |  |
| Ampulla of Vater | 1 | 2.7% | 0 | 0.0% | 2 | 0.8% |  | 1 | 1.8% | 0 | 0.0% | 4 | 0.9% |  |
| Biliary tract | 0 | 0.0% | 1 | 5.6% | 12 | 5.0% |  | 5 | 9.1% | 4 | 7.8% | 26 | 6.1% |  |
| Bladder/urinary tract | 0 | 0.0% | 0 | 0.0% | 2 | 0.8% |  | 0 | 0.0% | 0 | 0.0% | 6 | 1.4% |  |
| Bone | 0 | 0.0% | 0 | 0.0% | 0 | 0.0% |  | 0 | 0.0% | 1 | 2.0% | 1 | 0.2% |  |
| Bowel | 0 | 0.0% | 1 | 5.6% | 11 | 4.5% |  | 5 | 9.1% | 7 | 13.7% | 29 | 6.8% |  |
| Breast | 6 | 16.2% | 4 | 22.2% | 36 | 14.9% |  | 9 | 16.4% | 5 | 9.8% | 81 | 19.1% |  |
| Cervix | 0 | 0.0% | 0 | 0.0% | 1 | 0.4% |  | 1 | 1.8% | 2 | 3.9% | 9 | 2.1% |  |
| CNS/Brain | 0 | 0.0% | 0 | 0.0% | 4 | 1.7% |  | 0 | 0.0% | 0 | 0.0% | 8 | 1.9% |  |
| Esophagus/stomach | 1 | 2.7% | 0 | 0.0% | 5 | 2.1% |  | 3 | 5.5% | 2 | 3.9% | 15 | 3.5% |  |
| Eye | 0 | 0.0% | 0 | 0.0% | 0 | 0.0% |  | 0 | 0.0% | 0 | 0.0% | 0 | 0.0% |  |
| Head and neck | 0 | 0.0% | 1 | 5.6% | 7 | 2.9% |  | 1 | 1.8% | 0 | 0.0% | 6 | 1.4% |  |
| Kidney | 0 | 0.0% | 0 | 0.0% | 0 | 0.0% |  | 0 | 0.0% | 0 | 0.0% | 0 | 0.0% |  |
| Liver | 0 | 0.0% | 0 | 0.0% | 0 | 0.0% |  | 0 | 0.0% | 0 | 0.0% | 2 | 0.5% |  |
| Lung | 0 | 0.0% | 0 | 0.0% | 2 | 0.8% |  | 1 | 1.8% | 0 | 0.0% | 7 | 1.6% |  |
| Other | 0 | 0.0% | 2 | 11.1% | 5 | 2.1% |  | 2 | 3.6% | 6 | 11.8% | 7 | 1.6% |  |
| Ovary/Fallopian tube | 22 | 59.5% | 3 | 16.7% | 102 | 42.1% |  | 3 | 5.5% | 0 | 0.0% | 29 | 6.8% |  |
| Pancreas | 1 | 2.7% | 0 | 0.0% | 17 | 7.0% |  | 12 | 21.8% | 11 | 21.6% | 55 | 12.9% |  |
| Penis | 0 | 0.0% | 0 | 0.0% | 0 | 0.0% |  | 0 | 0.0% | 0 | 0.0% | 0 | 0.0% |  |
| Peripheral nervous system | 0 | 0.0% | 0 | 0.0% | 0 | 0.0% |  | 0 | 0.0% | 0 | 0.0% | 1 | 0.2% |  |
| Peritoneum | 4 | 10.8% | 0 | 0.0% | 10 | 4.1% |  | 3 | 5.5% | 1 | 2.0% | 6 | 1.4% |  |
| Pleura | 0 | 0.0% | 0 | 0.0% | 2 | 0.8% |  | 0 | 0.0% | 0 | 0.0% | 1 | 0.2% |  |
| Prostate | 0 | 0.0% | 0 | 0.0% | 4 | 1.7% |  | 6 | 10.9% | 8 | 15.7% | 98 | 23.1% |  |
| Skin | 1 | 2.7% | 0 | 0.0% | 3 | 1.2% |  | 1 | 1.8% | 1 | 2.0% | 4 | 0.9% |  |
| Soft Tissue | 0 | 0.0% | 2 | 11.1% | 1 | 0.4% |  | 0 | 0.0% | 1 | 2.0% | 3 | 0.7% |  |
| Testis | 0 | 0.0% | 0 | 0.0% | 0 | 0.0% |  | 0 | 0.0% | 0 | 0.0% | 1 | 0.2% |  |
| Thymus | 0 | 0.0% | 0 | 0.0% | 2 | 0.8% |  | 1 | 1.8% | 0 | 0.0% | 0 | 0.0% |  |
| Thyroid | 0 | 0.0% | 0 | 0.0% | 0 | 0.0% |  | 1 | 1.8% | 0 | 0.0% | 0 | 0.0% |  |
| Uterus | 1 | 2.7% | 4 | 22.2% | 13 | 5.4% |  | 0 | 0.0% | 2 | 3.9% | 25 | 5.9% |  |
| Vulva/vagina | 0 | 0.0% | 0 | 0.0% | 1 | 0.4% |  | 0 | 0.0% | 0 | 0.0% | 0 | 0.0% |  |
