# Supplementary material for: Pathogenic Germline Variants in BRCA1/2 and p53 Identified by Real-world Comprehensive Cancer Genome Profiling Tests in Asian Patients
Source: Cancer Res Commun. 2023 Nov 14;3(11):2302–11. doi: 10.1158/2767-9764.CRC-23-0018 (PMC10644847; doi:10.1158/2767-9764.CRC-23-0018)
Supplement: Figure S1 — The time-related changes in the OncoGuideTM NCC oncopanel system [file crc-23-0018-s05.docx]

**Supplementary Figure S1.**

The time-related changes in the OncoGuide^TM^ NCC oncopanel system (NOP) and differences in the genes for disclosing cancer susceptibility genes (CSGs) to patients in the panel before and after the revision.
