# Supplementary material for: Pathogenic Germline Variants in BRCA1/2 and p53 Identified by Real-world Comprehensive Cancer Genome Profiling Tests in Asian Patients
Source: Cancer Res Commun. 2023 Nov 14;3(11):2302–11. doi: 10.1158/2767-9764.CRC-23-0018 (PMC10644847; doi:10.1158/2767-9764.CRC-23-0018)
Supplement: Figure S2 — Distribution of variant allele frequency [file crc-23-0018-s06.docx]

**Supplementary Figure S2.**

Distribution of variant allele frequency for pathogenic germline variants (PGVs) in the OncoGuide^TM^ NCC oncopanel system (NOP). Cancer susceptibility genes (CSGs) that were subject to PGV reporting in NOP Ver.2 were analyzed using only the NOP Ver.2 data, and CSGs that were subject to PGV reporting in NOP Ver.1 were analyzed using the NOP data for the entire period. Numbers in the graph indicate the number of variants.
